# Melkersson-Rosenthal Syndrome: A Case Report With a Bipolar Perspective

**DOI:** 10.7759/cureus.35955

**Published:** 2023-03-09

**Authors:** Tina Kana, Ahmed Kawamj, Zaineb Shamim, Harish Rengarajan

**Affiliations:** 1 Internal Medicine, Touro College of Osteopathic Medicine, New York City, USA; 2 Internal Medicine, New York Medical College, Passaic, USA; 3 Internal Medicine, NYU Langone Health, New York City, USA

**Keywords:** bipolar disorders, fissured tongue, facial palsy, orofacial edema, melkersson-rosenthal syndrome

## Abstract

Melkersson-Rosenthal syndrome (MRS) is a rare neuro-mucocutaneous condition that presents with orofacial swelling, facial paralysis, and a fissured tongue. These classic triad of symptoms, however, very rarely present simultaneously. The symptoms are often seen alone or in pairs and appear at any stage in life. Although the etiology of this condition is unknown, various contributing factors have been suggested including infections, immune deficiencies, stress, and genetic predispositions. We present a case of a 23-year-old female patient who has a longstanding history of MRS, anxiety, and depression, and who attempted to overdose on prescription medications due to suicidal ideations.

## Introduction

Melkersson-Rosenthal syndrome (MRS) is a rare clinical syndrome defined by a classic triad of symptoms which includes recurrent facial nerve palsy, orofacial edema, and furrowing of the tongue [1]. Diagnosis can be extremely difficult given that patients are more frequently monosymptomatic or oligosymptomatic [2]. MRS was first documented by Melkersson in 1928 when he published a case of a 35-year-old female presenting with orofacial edema and recurrent facial palsy [3]. The third symptom, lingua plicata or fissured tongue, was added by Rosenthal in 1931 [4].

The reported incidence of MRS is approximately 0.08% within the general population and is more frequently diagnosed in young adults between the ages of 20 and 40 years [5]. The precise etiology is unknown, it is speculated that genetic predispositions, infections, autoimmune deficiencies, food intolerance, and stress may play a role [1]. Diagnosis is made directly if the full triad is present, however, if one or two symptoms are present, a skin biopsy should be performed which histologically demonstrates granulomatous cheilitis [6]. Additional features include non-caseating granulomas, Langerhan-type giant cells with multiple nuclei, lymphedema, and fibrosis [7]. The aim of this report was to create awareness of MRS in clinical practice as well as to describe the psychological facets of the condition.

## Case presentation

A 23-year-old female patient with a reported history of Melkersson-Rosenthal syndrome and bipolar disorder was brought to the ED after being found by her grandmother at home unresponsive with empty bottles of clonazepam and gabapentin next to her. History was obtained from the patient’s sister, a nurse, due to her altered mental status. The patient has a longstanding psychiatric history of anxiety and depression and was discharged three weeks prior from an inpatient psychiatry hospital where she was admitted for suicidal ideations and diagnosed with bipolar disorder. Her BMI at the time of admission was 34.44 kg/m^2^, and prior to this episode, the patient reported a five-week period of hypersomnia, a diminished ability to concentrate as well as feelings of worthlessness and guilt. The patient has a history of suicidal ideations and had previously partially acted on these ideations by grabbing a knife and mentioning she would hurt herself but eventually putting it down. She has also reportedly grabbed handfuls of pills and put them in her mouth before spitting them out in front of a family member. The patient was being followed at an intensive outpatient psychiatry program but was switched a few days ago to a different program. There was no history of bipolar disorder or any other psychiatric illnesses within her family. According to the sister, the patient is currently taking lurasidone, mirtazapine, clonazepam, and gabapentin. The prescription for clonazepam and gabapentin was refilled for a 30-day supply one week ago. Given the bottles were found empty, the sister assumed the patient had ingested a three-week supply of these medications. There was no reported seizure-like activity when the patient was found. The medications were taken between 4 and 6 AM, however, this is a rough estimate as the patient was not found until noon by her grandmother. Her sister also reported that the patient was with her boyfriend until approximately 4 AM and reportedly had a mental breakdown the night prior. The initial laboratory findings were unremarkable and imaging findings included a normal chest x-ray and CT of the head. Urinalysis was negative for amphetamines, benzodiazepines, cannabinoids, opiates, and barbiturates. The patient was admitted to the progressive case unit for closer monitoring, observation, and evaluation by the psychiatry team.

## Discussion

MRS is an extremely rare disease and there is little evidence of racial distribution [8]. However, it does appear to affect females three times as often as males [9]. The classic signs of lip swelling, fissured tongue, and facial paralysis are only seen in 8-18% of patients affected by MRS, whereas the remaining cases are monosymptomatic or oligosymptomatic [6]. As a result, diagnosis is very challenging [10]. The differential diagnosis of MRS includes a vast array of heterogenous conditions, primarily represented by granulomatous disorders that include Crohn's disease, sarcoidosis, Wegener’s vasculitis, and amyloidosis as well as a plethora of infections, such as Bell’s palsy, orofacial herpes, contact dermatitis, and allergic reactions [11]. Moreover, monosymptomatic variants of MRS that involve facial or lip swelling firmly be mistaken for angioedema [12]. The patient in this particular case had all three clinical findings, confirming the clinical diagnosis.

There is relatively little information regarding MRS and the psychological clinical manifestations' impact of the syndrome on patients [13]. Apart from the three classic features of MRS, migraines and dizziness have been reported in approximately half of MRS patients [14]. More sporadic neurological symptoms which have also been recorded include, deafness, difficulty swallowing, hypogeusia, aphthous ulcers, excessive lacrimation, and visual disturbances indicating involvement of other cranial nerves, such as the trigeminal, olfactory, auditory, and hypoglossal nerves [15]. More importantly, recurrent episodes of MRS have the potential to lead to personality changes, anxiety, and depression [16].

The current clinical case report features how significant emotional states can act as triggers in Melkersson-Rosenthal syndrome. In this particular case, the patient had an exacerbation of symptoms including mild upper-lip swelling and facial palsy. Upon further questioning, the patient revealed she was diagnosed with major depression at the age of 14 years after her first attempt at suicide. She had attempted suicide by overdosing on acetaminophen. She also admitted to cutting behaviors from a young age with the last episode being one year prior. The patient was having trouble in her relationship with her boyfriend prior to the current suicide attempt and was smoking marijuana, although the drug screen was negative. It is possible to get a negative screen, however, if she was using synthetic marijuana. Additionally, she was telling the grandmother in the emergency department “I should have taken more” several times.

A previous study by Alves et al. explored the psychosomatic aspects of the syndrome and evaluated the benefits of a brief psychotherapeutic process to reintegrate the bio-psycho-social functioning of a 26-year-old female patient with MRS suffering from depression. In the study, the patient had MRS relapses when she found out she would be separated from her boyfriend for a significant amount of time. She was anxious with depressive mood symptoms. She continued to have small relapses associated with minor stresses, such as work overload or sleep deprivation [17].

The patient in the current case had claimed that she was unable to sleep and had anxiety over the fight with her boyfriend. This led her to smoke marijuana, which did not help her sleep and ultimately she ingested more of her medications. The current clinical reports the importance of accounting for affective phenomena which can act as triggers in MRS. Several stressors have been identified for certain conditions, such as psoriasis in Crohn’s disease [18]. Although the underlying mechanisms that explain the association between psychological stressors and MRS are unknown, they are likely mediated by psychological, neurological, and immunological processes [8].

There is currently no specific treatment for MRS [12]. Since there appears to be a significant role of abnormal immune function, immune dysregulation, and allergic tendencies in patients, corticosteroids have served as the mainstay of treatment [16]. No randomized trials have been conducted to determine the type of corticosteroids to be used or the length of time that they should be used [16]. Currently, oral corticosteroids are being used for one week and tapered over a two-week period [16]. In several cases, high-dose methylprednisolone has been used [16]. Orofacial edema is treated with intralesional triamcinolone acetonide [16]. In refractory cases, second-line immunosuppressants such as methotrexate and thalidomide have been used [19].

## Conclusions

Melkersson-Rosenthal syndrome is a rare disorder that typically presents with a triad of symptoms that includes facial palsy, orofacial swelling, and a fissured tongue. Other granulomatous disorders and angioedema should be considered in the differential diagnosis, due to signs and symptoms overlapping. The current case report offers a new perspective on the treatment of Melkersson-Rosenthal syndrome patients by opening the possibility of providing psychiatric and psychological therapeutic interventions to aid in preventing exacerbations and relapses in treatment. It demonstrates the importance of the role of affective phenomena acting as triggers in MRS. Psychiatric and psychological therapeutic interventions play an important role in the psychological state of the patient.
